# Physioxia Reprograms Glioblastoma Cells Enhancing Migration and Altering Therapeutic Sensitivity

**DOI:** 10.3390/cancers18162620

**Published:** 2026-08-14

**Authors:** Natasha Hockaden, Elise O’Herron, Dylan Zhou, Nicholas Downing, Jacob Kurlander, Margaret Heffernan, Scott Cooper, Angela Richardson

**Affiliations:** 1 Neurological Surgery, Indiana University, Indianapolis, IN 46202, USA; nhockade@iu.edu (N.H.); eoherron@iu.edu (E.O.);; 2 School of Medicine, Indiana University, Indianapolis, IN 46202, USA; 3 Simon Comprehensive Cancer Center, Indiana University, Indianapolis, IN 46202, USA; 4 Department of Biology, University of Notre Dame, Notre Dame, IN 46556, USA

**Keywords:** glioblastoma, physioxia, tumor microenvironment, cell migration, therapeutic resistance, PDGFRβ signaling, oxygen tension, patient-derived glioblastoma

## Abstract

Background/Objectives: Glioblastoma is an aggressive primary brain tumor that develops within a chronically low-oxygen microenvironment, yet most preclinical studies are performed under atmospheric oxygen conditions that poorly reflect in vivo physiology. This study investigated how sustained culture under physiological oxygen tension (physioxia) influences glioblastoma cell behavior, signaling, and therapeutic response. Methods: Multiple patient-derived glioblastoma models were cultured under normoxia (21% O_2_) or sustained physioxia (5% O_2_) for at least seven days before experimentation. Cell migration, proliferation, cell cycle distribution, expression of the epithelial-to-mesenchymal transition-associated transcription factor Slug (SNAI2), PDGFRβ-associated signaling, and sensitivity to 5-fluorouracil were evaluated using transwell migration assays, cell counting, flow cytometry, RT-qPCR, immunoblotting, and BrdU incorporation assays. To examine the effects of oxygen history, we established and maintained additional patient-derived cultures grown under physioxia. Results: Sustained physioxia consistently increased migration across all glioblastoma models while reducing proliferation in normoxia-adapted cell lines through increased G0/G1 cell cycle arrest. Physioxia significantly increased Slug expression in all models and enhanced PDGFRβ, AKT, and ERK phosphorylation in a cell line-dependent manner. Therapeutic sensitivity to 5-fluorouracil was altered, with physioxia conferring increased resistance in some glioblastoma models but not universally. Patient-derived cell lines cultured continuously under physioxia retained enhanced migratory capacity and exhibited increased proliferation compared to cells grown in normoxia, suggesting that prior oxygen exposure influences proliferative responses while the pro-migratory phenotype remains conserved. Conclusions: Physiological oxygen tension is a major regulator of glioblastoma cell behavior, influencing migration, proliferation, signaling, and therapeutic response. These findings demonstrate that conventional normoxic culture conditions can obscure biologically relevant phenotypes and support the consideration of physioxia in experimental design. Such technical changes may improve the physiological and translational relevance of preclinical glioblastoma research.

## Simple Summary

Glioblastoma is the most aggressive form of brain cancer. It can spread rapidly and develop resistance to therapy, making treatment difficult. Most in vitro systems culture glioblastoma cells in oxygen levels that are much higher than those found in vivo, which may limit how well these models reflect the disease. In this study, we demonstrated how culturing glioblastoma cells under physiologically relevant oxygen levels (physioxia) that closely resemble those in the brain can alter cellular phenotype. We found that these growth conditions consistently increased tumor cell migration while altering their growth, signaling pathways, and response to chemotherapy. We also demonstrate that previous exposure to oxygen can influence cellular behavior. These findings suggest that the use of physioxic culture conditions in laboratory studies could improve the accuracy of glioblastoma research and support the development of more effective therapies.

## 1. Introduction

Glioblastoma (GB) is the most aggressive primary malignancy of the central nervous system and is characterized by diffuse invasion, extensive cellular heterogeneity, and resistance to therapy [1]. Despite maximal surgical resection followed by radiotherapy and temozolomide, median survival remains approximately 15 months [1,2]. This highlights the need for therapeutic strategies that complement conventional treatment.

Increasing evidence suggests that combination therapies targeting multiple hallmarks of GB, including tumor metabolism, invasion, stemness, DNA damage repair, and therapeutic resistance, may improve treatment efficacy while limiting tumor adaptation [2,3,4]. Emerging therapeutic and adjuvant strategies underscore the need to identify molecular mechanisms driving GB progression and therapy resistance within the tumor microenvironment and physiologically relevant growth conditions. These insights may reveal novel vulnerabilities for future combination therapies.

A defining feature of GB is the presence of heterogeneous oxygen gradients that influence tumor cell signaling, proliferation, invasion, and therapeutic response [5,6,7]. Standard cell culture conditions expose cells to atmospheric oxygen (~21% O_2_), which substantially exceeds oxygen levels present in normal brain tissue and GB tumors [8,9]. Physiological oxygen tension in the brain typically ranges from 2–8% O_2_, whereas GB tumors frequently contain regions with oxygen levels approaching 1% due to abnormal vasculature and rapid tumor growth [6]. Compared to conventional ambient air cell culture, cultures grown under physioxia (~5% O_2_) more closely resemble the oxygen conditions experienced by GB cells in vivo.

Most studies examining oxygen-dependent tumor biology have focused on acute exposure to hypoxia, lasting from minutes to 48 h [10]. Although these systems have defined critical hypoxia-responsive pathways, they primarily capture transient stress responses rather than stable adaptations associated with chronic low-oxygen exposure in vivo [6,8,9,10]. Reduced oxygen conditions within a tumor have been shown to influence GB cellular phenotypes and response to therapy [6,8,11]. Long-term culture under physioxia is therefore required to model the sustained oxygen adaptation that occurs in situ rather than acute hypoxic stress. Prior studies assessing the effects of oxygen tension on GB have identified alterations in many cellular pathways. However, many groups examining “chronic hypoxia” often limit exposure to a 48–72 h incubation; routine cell passaging and experiments were typically performed in ambient air [12,13,14,15,16].

Work by Broxmeyer and colleagues demonstrated that physioxia induces robust changes in cellular behavior, including altered stemness, signaling, and cell cycle regulation [10,11,17,18,19]. These studies further showed that brief exposure to atmospheric oxygen can rapidly alter cellular phenotypes [11,19]. Such observations are particularly relevant to GB, which contains stem-like tumor populations associated with invasion, recurrence, and therapeutic resistance. In neural and endothelial cell systems, prolonged physioxic culture alters differentiation and stress responses, suggesting that conventional normoxic conditions may obscure physiologically relevant behavior [17,20,21,22,23].

Among the signaling pathways influenced by oxygen tension, p53 and PDGFRβ-associated signaling are of particular interest in GB. The tumor suppressor p53 regulates cellular responses to stress, including DNA damage and hypoxia [24,25]. TP53 mutations are common in GB and influence tumor progression, signaling responses, and therapeutic sensitivity [17]. Oxygen tension can modulate p53 activity in a context-dependent manner, with distinct effects observed in wild-type and mutant p53 backgrounds [26,27].

Platelet-derived growth factor receptor beta (PDGFRβ) and its downstream PI3K/AKT and MAPK/ERK signaling pathways are major regulators of GB proliferation, survival, cytoskeletal remodeling, and migration [17,28,29]. These pathways are sensitive to oxygen availability and redox states [17]. In other tumor systems, physioxia enhances PDGFRβ-dependent signaling and promotes migratory behavior through sustained activation of AKT and ERK [17]. Loss of p53 function can further amplify these signaling responses by relieving inhibition of AKT and ERK pathways under low-oxygen conditions [30,31].

Together, these observations identify oxygen tension as a major determinant of tumor cell behavior rather than a passive culture variable [10,11,17]. Modeling GB under chronic physioxia may provide a more accurate representation of in vivo signaling states, invasive behavior, and therapeutic response. In this study, we investigated how sustained physioxia (minimum of 7 days, with cells passaged and maintained at physiologic oxygen levels) influences GB migration, proliferation, cell cycle progression, and signaling compared to cells cultured in ambient air. We further examined the relationship between oxygen tension, p53 status, and PDGFRβ-associated AKT and ERK signaling to define the mechanisms regulating GB adaptation to the brain microenvironment.

## 2. Materials and Methods

### 2.1. Cell Culture and Materials

GBM43 (RRID: CVCL_E5GD) and GBM10 cells were obtained from the Mayo Clinic (Rochester, MN, USA). GBM001 cells were a generous gift from Dr. Michael Ivan from the University of Miami. Cells were cultured in 21% O_2_ for normoxia experiments. For physioxia experiments, cells were maintained at 5% O_2_ for at least 7 days prior to being utilized for any experiments. Experiments were carried out in tandem with the same passage number in normoxia and physioxia to reduce variability. AR022 and AR023 cells were obtained from patients undergoing surgical resection with informed consent for research use (IRB-04, 25737). Tumors were transported in a hypoxia chamber. Cells were isolated and maintained in either ambient air conditions containing 21% O_2_ (normoxia, using a typical biosafety cabinet) or physiological oxygen tension (physioxia, Biospherix Chamber (Parish, NY, USA) set to 5% O_2_). For cell maintenance and all experiments, plasticware, media, PBS, and drug solutions were acclimated for at least 24 h to the appropriate oxygen tensions. Once cells were fixed, downstream experiments were carried out in ambient air.

GBM43, GBM001, and GBM10 were cultured in DMEM/F12 (Cytiva SH30023.01, Marlborough, MA, USA) supplemented with 10% FBS (Corning 35015CV, Corning, NY, USA), 1% penicillin-streptomycin (Cytiva SV30079.01), 1% Sodium Pyruvate (Gibco 11360070, Thermo Fisher Scientific, Waltham, MA, USA), and 1% MEM Non-Essential Amino Acids (GIBCO 11140050) at 37 °C in 5% CO_2_.

AR022 and AR023 cells were cultured in DMEM/F12 (Cytiva SH30023.01) supplemented with 2.5% FBS (Corning 35015CV), 1% penicillin-streptomycin (Cytiva SV30079.01), 1% Sodium Pyruvate (Gibco 11360070), 1% MEM Non-Essential Amino Acids (GIBCO 11140050), B-27™ Supplement minus vitamin A (Invitrogen 12587010, Carlsbad, CA, USA), N2™ Supplement minus vitamin A (Invitrogen, 17502048), rhEGF, 20 ng/mL (R&D Systems, 236-EG, Minneapolis, MN, USA), and rhFGF, 20 ng/mL (R&D Systems, BT-FGFHS) at 37 °C in 5% O_2_. Newly established patient-derived GB cultures were maintained in serum-reduced, growth factor-supplemented medium to preserve viability and maintain characteristics of the parental tumor during early establishment. Previously established GB cell lines were adapted to serum-containing culture conditions before this study. Culture conditions therefore reflect optimized maintenance protocols for each model rather than the experimental variables introduced for comparison.

### 2.2. Proliferation

For proliferation/growth curve assays, cells were initially maintained in standard tissue culture flasks and routinely passaged at approximately 80% confluence in each oxygen condition. Cells were seeded at a density of 10,000 cells per well in 24-well tissue culture plates under the indicated oxygen conditions. Cells were maintained in culture without passaging throughout the duration of the proliferation assay to allow longitudinal assessment of population expansion. Culture medium was refreshed every 3 days to maintain adequate nutrient availability and optimal culture conditions. Separate wells were designated for each experimental time point. Individual wells were harvested at the indicated time points by trypsinization and resuspended in fresh culture medium. Total cell number and cell viability were determined using trypan blue exclusion and manual counting with a hemocytometer. Each time point represents an independent terminal measurement from separate wells rather than repeated measurements of the same well or measurements from serially passaged cultures. Routine passaging at approximately 80% confluence was performed only for maintenance and expansion of stock cultures and was not performed during the proliferation assays. Cell cultures were allowed to continue growing throughout the assay and were not harvested specifically upon reaching 80% confluence; this was done to characterize the full growth trajectory under each oxygen condition. As cultures approached high density or confluence, population expansion was expected to become limited by density-dependent growth and available nutrients, and later time points were therefore interpreted as reflecting overall changes in cell population expansion rather than proliferation rate alone.

### 2.3. Chemotherapy Response

For chemotherapy response experiments, cells were seeded into 96-well tissue culture plates at densities optimized for each cell line (GBM43: 3000 cells/well; GBM10: 3000 cells/well; GBM001: 5000 cells/well; AR022: 2000 cells/well; AR023: 2000 cells/well). Cells were allowed to adhere overnight under their respective experimental oxygen conditions prior to drug treatment. For experiments conducted under physioxia, cells and culture plates were maintained under 5% O_2_ throughout the experimental period. All media and reagents used for treatment were equilibrated to the appropriate oxygen conditions prior to addition to the cells. Cells were treated with 5-fluorouracil (5-FU; Thermo Scientific, A13456.06) at the indicated concentrations, diluted in the standard culture medium specific to each cell line. Vehicle-treated cells maintained under the corresponding oxygen condition served as controls. Cells were exposed to 5-FU for 48 h under the appropriate oxygen conditions, after which cell proliferation was assessed by measuring DNA synthesis using a BrdU ELISA kit (Abcam, Cambridge, UK, ab126556), according to the manufacturer’s protocol. BrdU labeling times were optimized for each cell line (4 h for GBM43 and GBM10; 20 h for GBM001, AR022, and AR023). Following BrdU labeling, absorbance was measured at 450 nm, with each well read in triplicate. Background signal from no-cell control wells was subtracted from experimental measurements. BrdU incorporation was then normalized to the mean absorbance of vehicle-treated control wells maintained under the same oxygen condition. Normalized values represent relative proliferative activity following 5-FU exposure. Each experimental condition was evaluated using technical replicate wells, which were averaged within each independent biological experiment prior to statistical analysis.

### 2.4. Cell Cycle

Cells were cultured under standard conditions at the appropriate oxygen tension for each cell line. The following methods were performed under both oxygen tensions in tandem to minimize variability. GBM43, GBM10, and GBM001 cells were seeded in 10-cm tissue culture dishes at a density of 300,000 cells per dish. AR022 and AR023 cells were seeded in T180 flasks at a density of 600,000 cells per flask. Seeding densities were optimized for each cell line to achieve 60–70% confluence while allowing continued cell growth at the time of cell collection, rather than analyzing cells that had reached overconfluence. Cells were harvested at time points corresponding to the divergence of normoxic and physioxic growth curves for each cell line (day 3 for GBM43; day 7 for GBM10 and GBM001). Harvest time points were selected to identify changes in cell cycle distribution at the time of growth divergence while ensuring that cells cultured under one oxygen tension were not more confluent than those cultured under the other at the time of collection. Cells were fixed in 10% neutral buffered formalin and stained with FxCycle™ Far Red Stain (Invitrogen, F10348) according to the manufacturer’s protocol. Flow cytometry analysis was performed using 633/5 nm excitation and emission. Cell cycle distribution was determined by DNA content. The G0/G1, S, and G2/M phases were identified by histogram analysis.

### 2.5. RT-qPCR

For gene expression analysis, cells were maintained under the indicated oxygen conditions until the designated experimental endpoint. To minimize exposure to atmospheric oxygen prior to sample collection, cells cultured under 5% O_2_ were harvested directly within the controlled oxygen chamber. Cells were collected by trypsinization, and cell pellets were recovered and removed from the chamber for immediate RNA isolation. Cell pellets were lysed on the benchtop using the RNeasy^®^ Mini Kit (Qiagen, Venlo, The Netherlands, 74106) according to the manufacturer’s protocol. RNA concentration and purity were assessed using a NanoDrop spectrophotometer (Thermo Fisher Scientific), and equivalent amounts of RNA were reverse transcribed using the High-Capacity RNA-to-cDNA Kit (Applied Biosystems, Foster City, CA, USA, 4387406) according to the manufacturer’s instructions. Quantitative real-time PCR (RT-qPCR) was performed using SYBR Green master mix (Applied Biosystems, A25742) and gene-specific primers on a QuantStudio 6 Real-Time PCR System (Applied Biosystems, RRID: SCR_020239). Gene expression was normalized to GAPDH, and relative transcript expression was determined using the appropriate comparative Ct method. Primer sequences used were as follows: GAPDH forward 5′-TCAAGGCTGAGAACGGGAAG-3′ and reverse 5′-CGCCCCACTTGATTTTGGAG-3′; and SNAI2 (Slug) forward 5′-TCGGACCCACACATTACCTT-3′ and reverse 5′-TGACCTGTCTGCAAATGCTC-3′.

### 2.6. Immunoblotting

For immunoblotting experiments, cells were maintained under the indicated oxygen conditions until the designated experimental endpoint. To minimize exposure to atmospheric oxygen prior to sample collection, cells cultured under 5% O_2_ were harvested directly within the controlled oxygen chamber. Cells were collected by trypsinization, and cell pellets were recovered and removed from the chamber for immediate protein extraction on ice. Immunoblotting was performed using standard sodium dodecyl sulfate polyacrylamide gel electrophoresis techniques with RIPA buffer (50 mM Tris, 150 mM NaCl, 1 mM EDTA, 1% NP-40, 1% sodium deoxycholate, 1% SDS) used to lyse cells as previously described [32]. Antibodies used for immunoblotting included Histone 3 (Cell Signaling Technology 9715, Danvers, MA, USA, RRID: AB_331563), pPDGFRβ (Y751) (Cell Signaling Technology 4549, RRID: AB_1147704), PDGFRβ (Cell Signaling Technology 3162, RRID: AB_331111), pAKT (S473) (Cell Signaling Technology 4060, RRID: AB_2315049), AKT (Cell Signaling Technology 4691, RRID: AB_915783), pERK (T202/Y204) (Cell Signaling Technology 9101, RRID: AB_331646), ERK (Cell Signaling Technology 9102, RRID: AB_330744), P53 (Cell Signaling Technology 9282, RRID: AB_331476), pP53 (S15) (Cell Signaling Technology 9284, RRID: AB_331464), β-catenin (Cell Signaling Technology 9562, RRID: AB_331149), and pβ-catenin (S675) (Cell Signaling Technology 4176, RRID: AB_1903923).

### 2.7. Transwell Migration

For transwell migration assays, cells were maintained under the indicated oxygen conditions prior to and throughout the migration assay. Cells were serum-starved for 18 h under the appropriate experimental oxygen tension before initiating the assay. Migration was assessed using ThinCert™ Boyden chamber inserts (Greiner One, Kremsmünster, Austria, 662638) placed in 24-well plates. Following serum starvation, 50,000 cells were seeded into each insert in serum-free culture medium. For experiments conducted under 5% O_2_, cells were harvested and prepared for plating under the controlled oxygen conditions. All inserts, plates, and media were equilibrated to the appropriate oxygen tension before beginning the assay. Complete growth medium containing 10% FBS for GBM001, GBM10, and GBM43 cells or 2.5% FBS for AR022 and AR023 cells was added to the lower chamber to serve as a chemoattractant. Cells were plated in triplicate technical replicate inserts for each experimental condition and allowed to migrate for 24 h under the indicated oxygen conditions. Inserts were stained and analyzed as previously described [33].

### 2.8. Statistical Analysis

All experiments were performed using three independent biological replicates. Where applicable, each biological replicate included three technical replicates, which were averaged within each independent experiment prior to statistical analysis. Statistical analyses were performed using the biological replicate as the experimental unit (N = 1, n = 3), and technical replicates were not treated as independent biological observations. Reported *p*-values thus reflect the consistency of the observed responses across independent biological experiments rather than an inflation of sample size resulting from technical replication. All quantitative data are presented as mean ± standard error of the mean (SEM), unless otherwise indicated. Statistical analyses were performed using GraphPad Prism (version 10.4.2, San Diego, CA, USA). The number of biological replicates (independent experiments or patient-derived cell lines) and technical replicates for each assay are specified in the corresponding figure legends. For comparisons between two groups (e.g., normoxia versus physioxia), unpaired two-tailed Student’s *t* tests were used when data were normally distributed and variances were comparable. For experiments involving more than two groups or multiple conditions (e.g., dose–response analyses, or comparisons across multiple cell lines), two-way analysis of variance (ANOVA) was performed as appropriate. When ANOVA revealed a significant main effect or interaction, post hoc multiple-comparison testing was conducted using Tukey’s test. Transwell migration assays, proliferation assays, cell cycle distributions, gene expression analyses, and immunoblot quantifications were analyzed using biological replicates as the unit of analysis. For flow cytometry-based cell cycle analysis, percentages of cells in each phase (G0/G1, S, and G2/M) were compared between oxygen conditions using unpaired two-tailed *t* tests or ANOVA, depending on the number of groups analyzed. For chemotherapeutic response assays, dose–response curves were generated and percent survival was calculated relative to untreated controls for each oxygen condition. Statistical comparisons between normoxia and physioxia at individual drug concentrations were performed using two-way ANOVA with multiple-comparison correction. No curve fitting-based IC_50_ comparisons were performed unless explicitly stated. RT-qPCR data were analyzed using the ΔΔCt method, with expression levels normalized to housekeeping genes and presented relative to normoxic controls. Log-transformed ΔCt values were used for statistical testing to satisfy normality assumptions. Immunoblot densitometry was performed using ImageJ v1.54p or equivalent software. Phosphorylated protein levels were normalized to total protein or loading controls as indicated. Normalized values were analyzed using unpaired *t* tests or ANOVA as appropriate. All statistical tests were two-sided, and *p*-values < 0.05 were considered statistically significant. Exact *p*-values and statistical tests used are reported in the figure legends. No statistical methods were used to predetermine sample size, but sample sizes are consistent with those commonly reported in the field. No data was excluded from analysis unless pre-specified experimental quality control criteria were not met. Generative Artificial Intelligence (GenAI) Statement: The authors declare that no generative artificial intelligence (GenAI) tools were used in the conception, design, data collection, data analysis, interpretation of results, or preparation of figures.

## 3. Results

### 3.1. Physioxia Enhances Transwell Migration of Glioblastoma Cells

**Figure 1:**
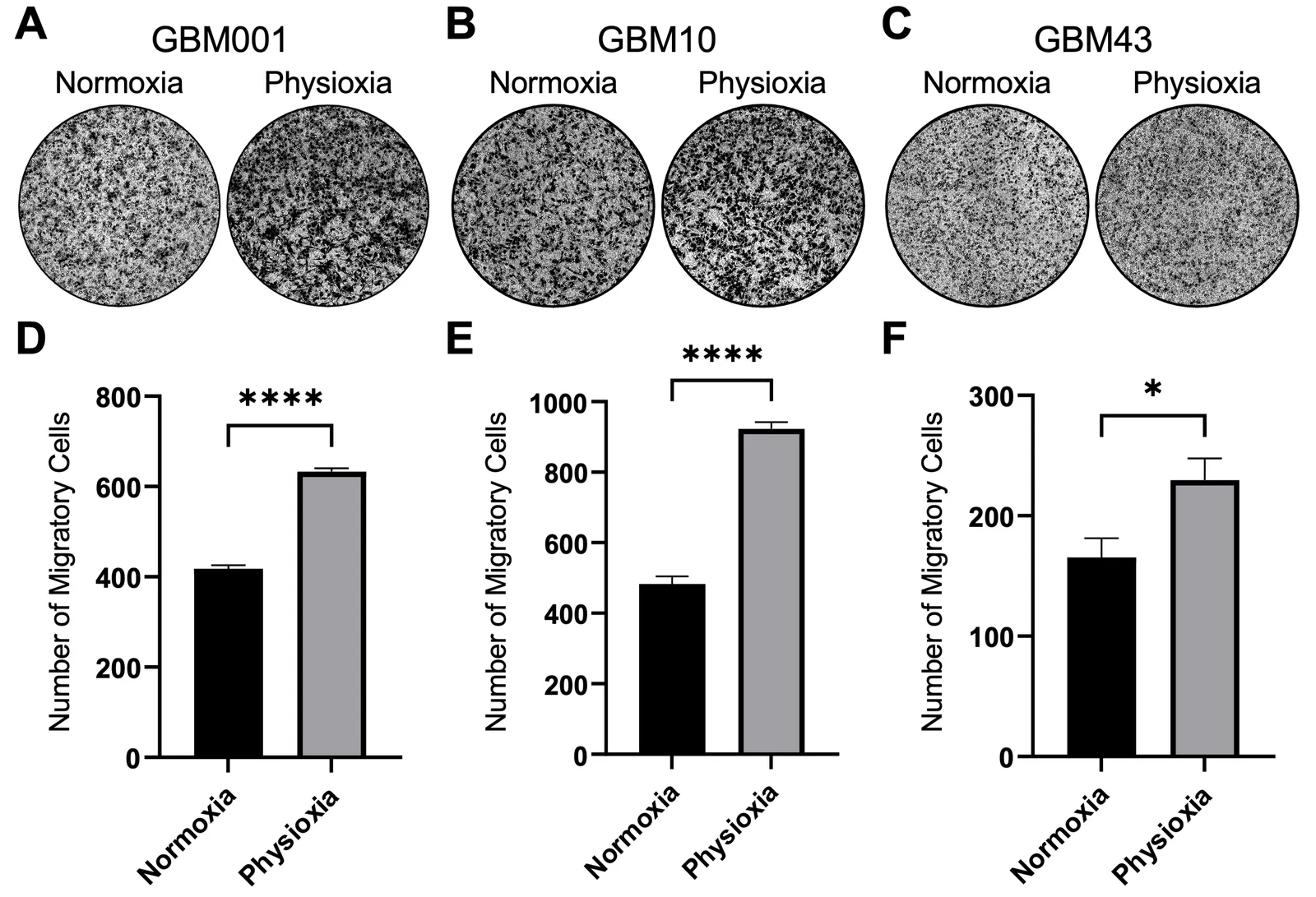
Physioxia enhances migratory capacity in glioblastoma cells. (**A**–**C**) Representative images of migrated (**A**) GBM001, (**B**) GBM10, and (**C**) GBM43 cells following a 24-h Transwell (Boyden chamber) migration assay performed under normoxic (21% O_2_) or physioxic (5% O_2_) conditions. (**D**–**F**) Quantification of migrated cells demonstrating a significant increase in migration under physioxia compared with normoxia in (**D**) GBM001, (**E**) GBM10, and (**F**) GBM43 cells. For each independent experiment, cells were plated in triplicate technical replicate inserts for each oxygen condition, and technical replicates were averaged prior to statistical analysis. Data are presented as mean ± SEM from three independent biological experiments (n = 3). Statistical significance was determined by two-way ANOVA, with * *p* < 0.05 and **** *p* < 0.0001.

Physioxia has been increasingly recognized as an important regulator of tumor cell behavior [8,11]. Prior studies in multiple cancer types, including breast, prostate, colorectal, and pancreatic cancers, have demonstrated that culturing cells under physioxia (typically 3–5% O_2_) enhances migratory and invasive capacity compared with ambient air conditions [17,20,34]. These effects have been attributed to oxygen-sensitive signaling pathways that regulate cytoskeletal dynamics, extracellular matrix interactions, and motility-associated gene expression [35]. Acute hypoxia has been associated with increased GB cell migration and invasion through activation of hypoxia-inducible signaling pathways [6]; however, the impact of sustained physioxia on the migratory behavior of patient-derived GB cells remains incompletely characterized. To evaluate the effects of oxygen tension on GB cell motility, transwell migration assays were performed using GBM001, GBM10, and GBM43 cells cultured under ambient air/normoxia (~21% O_2_) or physioxia (5% O_2_). Across all three GB lines, exposure to physioxia resulted in a significant increase in migratory capacity compared with cells maintained under ambient oxygen conditions (Figure 1).

Quantitative analysis revealed that GBM001 cells exhibited a robust elevation in transwell migration under physioxia relative to ambient air (Figure 1D). Similarly, GBM10 cells demonstrated a marked increase in the number of migrated cells when cultured at 5% oxygen (Figure 1E). GBM43 cells also showed a significant enhancement in migration under physioxic conditions, indicating that the pro-migratory effect of physioxia is conserved across genetically distinct GB models (Figure 1F).

These findings demonstrate that GB cells grown under physioxic culture conditions present increased migration capacity, extending prior observations from other cancer types to patient-derived GB models. These observations also highlight oxygen tension as a key microenvironmental determinant of GB invasiveness. Given the pronounced increase in migratory behavior observed under physioxia, we next examined whether physioxia affects GB cell growth characteristics. The following section investigates the effects of physioxia versus ambient air on cell proliferation and cell cycle distribution to determine whether enhanced migration is confounded by concurrent changes in proliferative dynamics.

### 3.2. Physioxia Reduces Glioblastoma Cell Proliferation over Extended Culture

Physioxia has been reported to modulate tumor cell proliferation in a context-dependent manner [36]. Previous studies in breast cancer and other solid tumors have shown that culturing cells under physioxia can suppress proliferation while promoting invasive or stem-like phenotypes, suggesting a potential trade-off between migratory capacity and cell cycle progression [17,34]. These observations provided rationale for examining whether the increased migration observed under physioxia in GB cells was accompanied by altered proliferative behavior.

**Figure 2:**
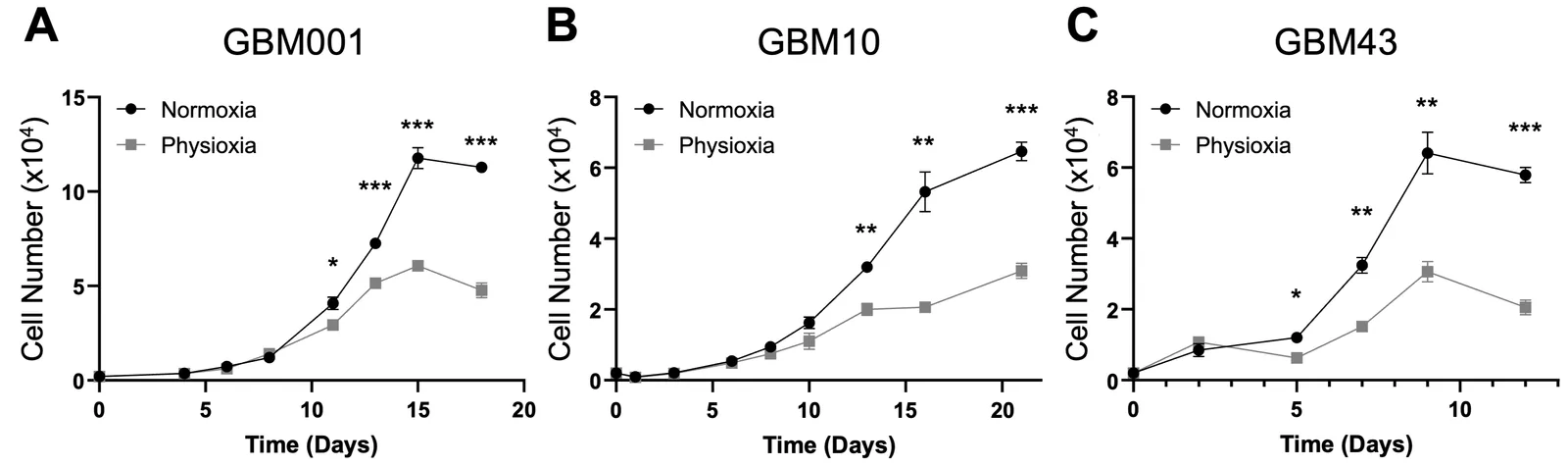
Physioxia reduces long-term population expansion of established glioblastoma cell models. (**A**–**C**) Longitudinal population growth of (**A**) GBM001, (**B**) GBM10, and (**C**) GBM43 cells cultured under normoxic (21% O_2_) or physioxic (5% O_2_) conditions over a 22-day period. For each experiment, cells were seeded at 10,000 cells per well in 24-well plates and maintained under the indicated oxygen conditions without passaging throughout the assay. Separate wells were harvested at each indicated time point, and viable cell numbers were determined by trypan blue exclusion and hemocytometer counting. Across the experimental period, all three established GB cell lines demonstrated reduced population expansion under physioxia compared with normoxia, with the magnitude and timing of the effect varying among cell lines. Data are presented as mean ± SEM from three independent biological experiments (n = 3), with each biological experiment performed using independent wells for each time point. Statistical significance between oxygen conditions at individual time points was determined using unpaired two-tailed Student’s *t*-tests, with * *p* < 0.05, ** *p* < 0.01, and *** *p* < 0.001.

To assess the effect of oxygen tension on long-term cell growth, GBM001, GBM10, and GBM43 cells were cultured under ambient air or physioxia, and total cell numbers were quantified over a 12-21 day period. As predicted for a finite-density culture system, cell numbers plateaued and subsequently declined at later time points. These observations are consistent with cultures approaching or exceeding optimal growth density. Thus, later time points likely reflect a combination of proliferative capacity, density-dependent growth limitation, and cell viability rather than proliferation rate alone. Across all three GB lines, chronic exposure to physioxia significantly reduced cell accumulation compared to cells cultured in ambient air, though the onset and magnitude of this effect varied by cell line (Figure 2).

In GBM001 and GBM10 cells, a decrease in proliferation under physioxia became evident by day 7 and persisted throughout the remainder of the culture period (Figure 2A,B). In contrast, GBM43 cells exhibited an earlier sensitivity to physiological oxygen tension, with significantly reduced cell numbers observed as early as day 3 under physioxic conditions (Figure 2C). These findings suggest that while physioxia consistently suppresses GB cell proliferation, the temporal response to reduced oxygen tension is cell line-dependent. These findings also illustrate the need for additional studies in sustained low oxygen levels to fully elucidate the impact of oxygen tension on phenotypic readouts.

Given the observed reduction in cell proliferation under physioxia, we next sought to determine whether these effects were associated with alterations in cell cycle progression. Based on prior reports linking physioxia to cell cycle regulation and quiescence, flow cytometry-based cell cycle analysis was performed. We chose time points corresponding to the onset of proliferation differences in each cell line to further characterize the mechanisms underlying physioxia-induced growth suppression.

### 3.3. Physioxia Induces Cell Cycle Arrest in Glioblastoma Cells

**Figure 3:**
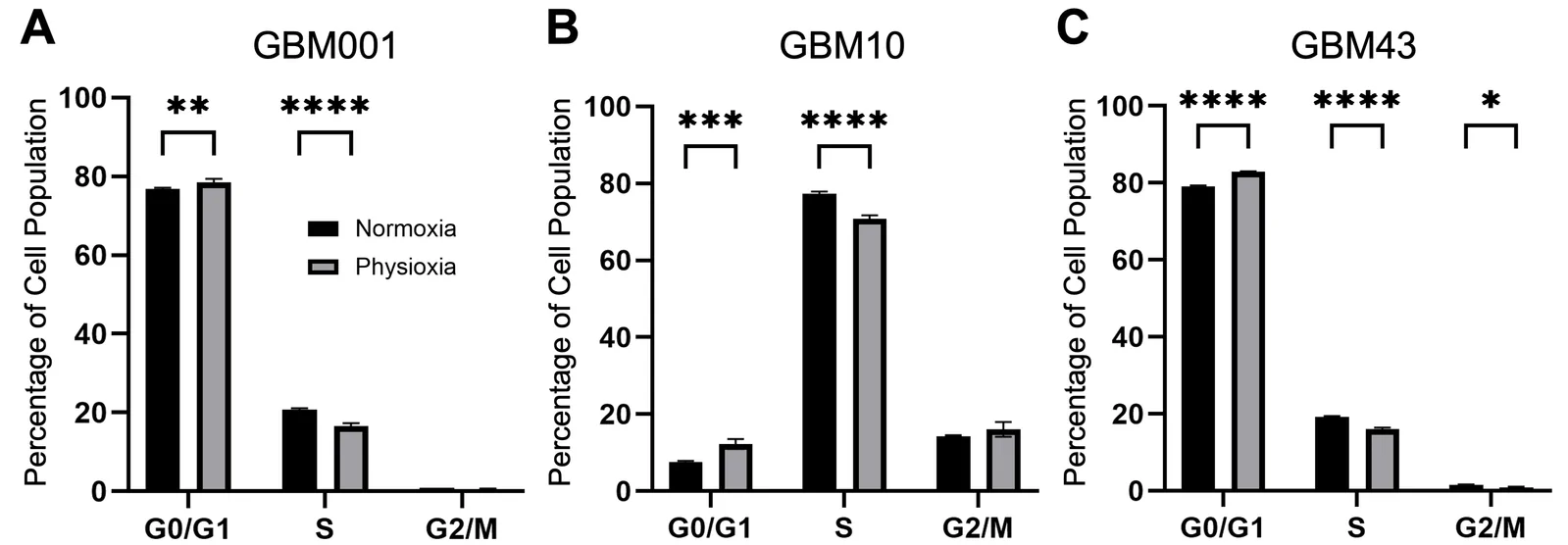
Physioxia alters cell-cycle distribution in glioblastoma cells. (**A**–**C**) Cell-cycle distribution was assessed by flow cytometry in (**A**) GBM001 cells at Day 7, (**B**) GBM10 cells at Day 7, and (**C**) GBM43 cells at Day 3 following culture under normoxic (21% O_2_) or physioxic (5% O_2_) conditions. Time points were selected based upon observed differences in population expansion among the individual cell lines. In GBM001 cells, physioxia increased the proportion of cells in the G0/G1 phase and was accompanied by a corresponding decrease in the S-phase population compared with normoxia. GBM10 cells cultured under physioxia exhibited increased G0/G1 accumulation and a reduction in the proportion of cells in S phase relative to normoxia. In GBM43 cells, physioxia increased the proportion of cells in G0/G1 while decreasing the fractions of cells in both S and G2/M phases compared with normoxia, consistent with a broader reduction in cell-cycle progression. These findings demonstrate oxygen-dependent changes in cell-cycle distribution but do not by themselves establish permanent or irreversible cell-cycle arrest. Data are presented as mean ± SEM from three independent biological experiments (n = 3). Statistical significance was determined by two-way ANOVA, with * *p* < 0.05, ** *p* < 0.01, *** *p* < 0.001, and **** *p* < 0.0001.

In GBM001 cells analyzed at Day 7, growth under physioxia resulted in a significant increase in the proportion of cells in the G0/G1 phase (Figure 3A). These results were accompanied by a corresponding decrease in S-phase cells compared with normoxia (Figure 3A). A similar pattern was observed in GBM10 cells at Day 7. An accumulation of cells in G0/G1 and a reduction in the proportion of cells in S phase was observed in GB culture under physioxic conditions (Figure 3B). These results suggest cell-cycle progression is reduced when cells are cultured in physiological oxygen conditions.

Cell-cycle analysis at day 3 revealed a pronounced shift in cell-cycle distribution for GBM43 cells cultured under physioxia (Figure 3C). Interestingly, GBM43 cells exhibited an earlier reduction in population expansion (Figure 2C). GBM43 cells cultured under physioxia displayed an increase in the G0/G1 population, accompanied by decreases in the proportions of cells in S phase and G2/M relative to normoxia (Figure 3C). These findings suggest a broader reduction in cell-cycle progression in GBM43 cells compared with GBM001 and GBM10 cultured in physioxic conditions. Although the observed G0/G1 accumulation is consistent with a quiescent-like phenotype, additional studies are needed to determine whether this state is reversible. Assessing the restoration of proliferation following re-exposure to growth-promoting conditions, together with senescence and cell death markers, would distinguish reversible quiescence from senescence or cytotoxicity.

Collectively, these findings indicate that physioxia is associated with reduced cell-cycle progression and decreased S-phase entry in GB cells. Cell line-specific differences influence the magnitude and timing of these effects. These changes occur in parallel with enhanced migratory capacity under physioxia (Figure 1), suggesting that physiological oxygen conditions may promote a shift in cellular behavior away from population expansion and toward motility. Such a proliferation-migration dichotomy has been previously described in GB and other invasive cancers. These observations may be associated with transitions toward mesenchymal or epithelial-to-mesenchymal transition (EMT)-like phenotypes. Given the opposing effects of physioxia on GB cell population expansion and migration, we next investigated whether physioxia promotes molecular programs associated with EMT and cell motility.

### 3.4. Physioxia Induces Slug Expression and Is Correlated with PDGFRβ-Associated Signaling Pathways in a Cell Line-Dependent Manner

Previous studies have linked physioxia to the induction of epithelial-to-mesenchymal transition (EMT)-associated transcription factor expression and activity, including Slug (SNAI2), in GB and other solid tumors [17]. Increased Slug expression under physioxia has been associated with enhanced migratory and invasive behavior, providing a mechanistic framework for the proliferation-migration dichotomy observed in GB [17]. Platelet-derived growth factor receptor beta (PDGFRβ) is a key regulator of GB cell motility. PDGFRβ can activate downstream signaling pathways involved in EMT, including AKT and ERK, and can influence Slug activity, an additional transcription factor driving tumor cell invasion [17]. Existing evidence suggests that PDGFRβ signaling can regulate Slug through post-transcriptional stabilization of the protein rather than increased transcription; however, these studies have been conducted under conventional normoxic culture conditions. We sought to evaluate whether chronic physioxia modulates Slug expression and PDGFRβ pathway activation in patient-derived GB cells.

**Figure 4:**
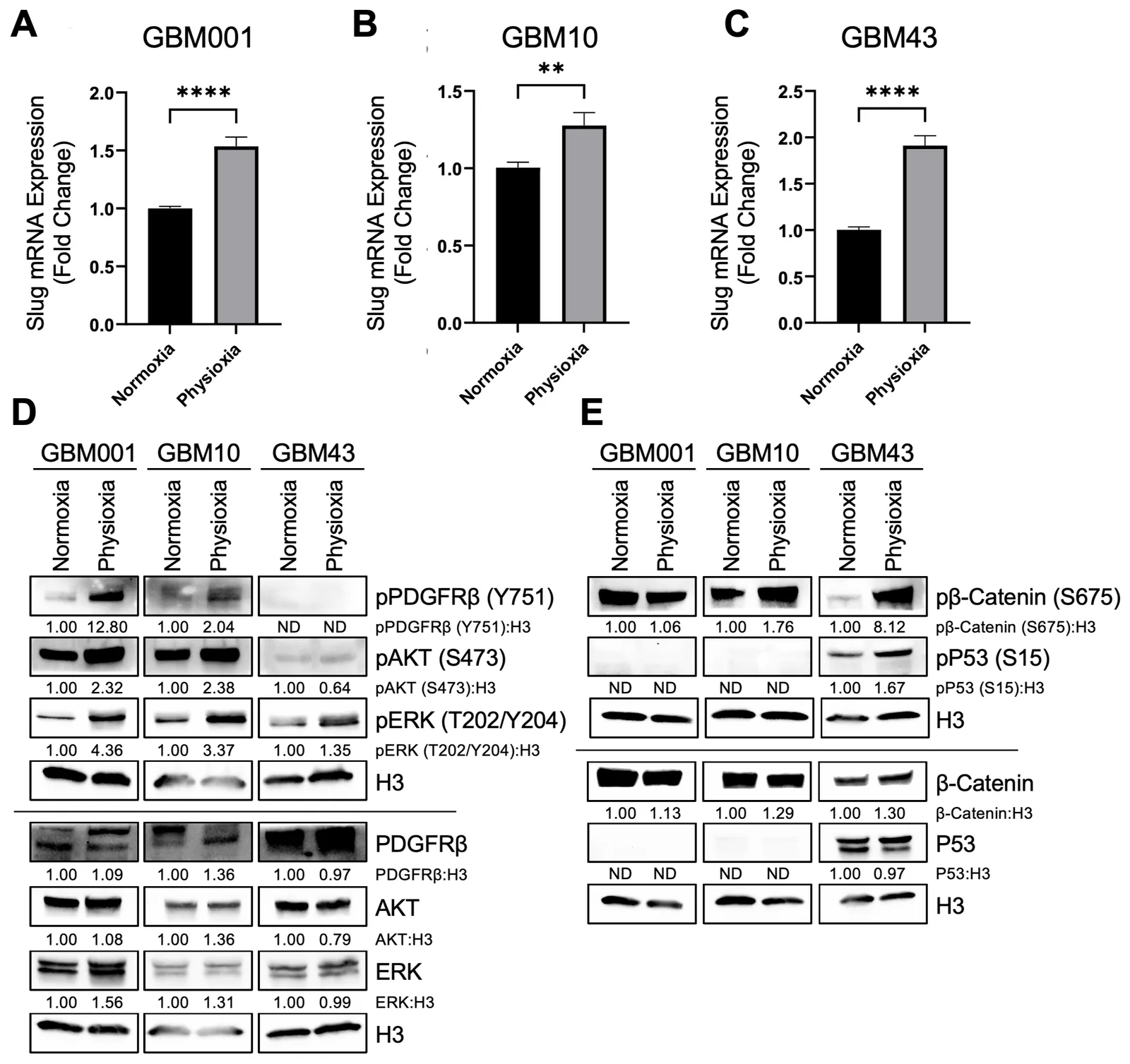
Physioxia increases SNAI2 (Slug) expression and alters signaling pathways associated with glioblastoma cell migration and plasticity. (**A**–**C**) Relative SNAI2 (Slug) mRNA expression was assessed by RT-qPCR in (**A**) GBM001, (**B**) GBM10, and (**C**) GBM43 cells following culture under normoxic (21% O_2_) or physioxic (5% O_2_) conditions. Physioxia was associated with increased SNAI2 transcript levels across all three GB cell lines compared with normoxia. (**D**) Representative immunoblot analysis of GBM001, GBM10, and GBM43 cells cultured under normoxic or physioxic conditions showing expression of phosphorylated PDGFRβ (Y751), total PDGFRβ, phosphorylated AKT (S473), total AKT, phosphorylated ERK (T202/Y204), and total ERK. Histone H3 was used as a loading control (Supplementary Materials Image S1). (**E**) Representative immunoblot analysis of GBM001, GBM10, and GBM43 cells cultured under normoxic or physioxic conditions showing total P53, phosphorylated P53 (S15), total β-catenin, and phosphorylated β-catenin (S675). Histone H3 was used as a loading control (Supplementary Materials Image S2). These findings demonstrate that physioxia is associated with transcriptional upregulation of SNAI2 and oxygen-dependent changes in the activation of signaling pathways involved in cell survival, migration, and cellular plasticity. Data are presented from three independent biological experiments (n = 3). Statistical significance for RT-qPCR data was determined using unpaired two-tailed Student’s *t*-tests, with ** *p* < 0.01 and **** *p* < 0.0001.

RT-qPCR analysis revealed a significant increase in Slug mRNA expression in GBM001, GBM10, and GBM43 cells cultured under physioxia compared to those grown in normoxic conditions (Figure 4A–C). This upregulation was observed across all three patient-derived GB lines. These results suggest that physioxia can induce transcriptional programs associated with mesenchymal identity and migration.

To determine whether physioxia-induced Slug expression was associated with upstream signaling pathways, immunoblotting was performed to assess PDGFRβ pathway activity. Protein lysates were probed for total and activated phosphorylated PDGFRβ (Y751), AKT (S473), and ERK (T202/Y204), with Histone H3 used as a loading control. In both GBM001 and GBM10 cells, physioxia resulted in increased phosphorylation of PDGFRβ at Y751, accompanied by elevated levels of phospho-AKT (S473) and phospho-ERK (T202/Y204), while total protein levels remained largely unchanged. These data indicate that physioxic culture conditions enhance phosphorylation of PDGFRβ signaling and its downstream activation of the AKT and ERK pathways in these cells (Figure 4D).

Despite an observed increase in Slug mRNA expression, GBM43 cells did not exhibit increased changes in PDGFRβ, AKT, or ERK phosphorylation under physioxia compared with normoxia. This lack of differential pathway activation may reflect constitutive signaling activity in GBM43 cells. Interestingly, GBM43 cells harbor a p53 mutation. Mutant p53 can activate growth factor signaling pathways independent of upstream receptor stimulation, including PI3K/AKT and MAPK/ERK. Such constitutive activation may limit the response to changes in oxygen tension (Figure 4D).

To further investigate the role of p53 signaling under physioxia, immunoblot analysis was performed for total p53, phospho-p53 (S15), total β-catenin and phospho-β-catenin (S675) in GBM001, GBM10, and GBM43 cells cultured under normoxic and physioxic conditions (Figure 4E). No detectable expression of total p53 or phospho-p53 (S15) was observed in GBM001 or GBM10 cells under either oxygen condition. It is important to note that GBM10 cells do not display a nonsynonymous/missense variant of P53. GBM43 cells demonstrated increased phospho-p53 (S15) and phospho-β-catenin (S675) levels under physioxia relative to normoxia. Given that GBM43 harbors a known p53 mutation, the observed increase in phospho-p53 may reflect stabilization and accumulation of mutant p53 protein rather than activation of canonical wild-type p53 tumor suppressor signaling [30,37]. Mutant p53 proteins frequently exhibit prolonged protein half-life and aberrant phosphorylation patterns that contribute to oncogenic gain-of-function activities, including enhanced survival, invasion, and adaptation to microenvironmental stress [30]. β-catenin signaling has been linked to EMT-associated transcriptional programs and invasive behavior in GB [38]. Increased phosphorylation of β-catenin at S675 suggests physioxia results in the activation of pro-survival and pro-migratory signaling pathways in GBM43 cells [38]. Together, these findings support a model in which physioxia promotes migration in GBM43 cells through mutant p53-associated signaling mechanisms distinct from a PDGFRβ-AKT-ERK axis present in GBM001 and GBM10 cells.

These results suggest that physioxic growth conditions can induce EMT-associated transcriptional changes across multiple GB cell lines, while PDGFRβ-AKT-ERK phosphorylation signaling occurs in a cell line-specific manner. In GBM001 and GBM10 cells, enhanced PDGFRβ pathway activity under physioxia provides a potential mechanistic link between increased Slug expression and the pro-migratory phenotype observed under physioxia. It is possible GBM43 cells rely on alternative or constitutive activation of signaling mechanisms to support physioxia-induced migration.

### 3.5. Physioxia Confers Cell Line-Specific Resistance to 5-Fluorouracil in Glioblastoma Cells

Physioxia can influence therapeutic response in GB and other solid tumors, with multiple studies demonstrating increased resistance to cytotoxic agents under physioxia or hypoxia [17,20,34]. Reduced oxygen levels promote drug tolerance by altering cell cycle dynamics, enhancing the DNA damage response, and activating survival pathways [39]. Based upon these observations, we sought to examine whether the physioxia-induced phenotypic changes observed in GB cells were associated with altered sensitivity to chemotherapy.

**Figure 5:**
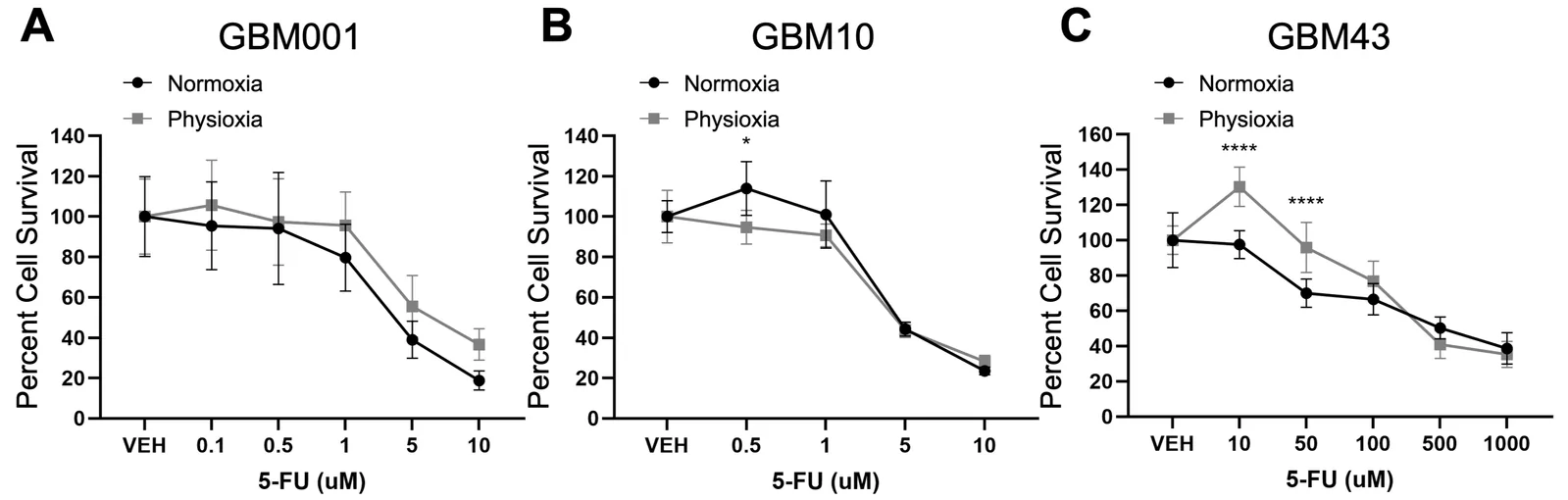
Physioxia differentially modulates glioblastoma cell responses to 5-fluorouracil (5-FU). (**A**–**C**) GBM001 (**A**), GBM10 (**B**), and GBM43 (**C**) cells were exposed to increasing concentrations of 5-fluorouracil (5-FU) for 48 h under normoxic (21% O_2_) or physioxic (5% O_2_) conditions. Cellular proliferative activity following 5-FU exposure was assessed by BrdU incorporation and expressed relative to vehicle-treated controls maintained under the corresponding oxygen condition. All three GB cell lines demonstrated dose-dependent reductions in BrdU incorporation with increasing 5-FU concentrations under both oxygen conditions, consistent with a concentration-dependent inhibition of DNA synthesis. GBM43 cells exhibited increased relative BrdU incorporation under physioxia at lower 5-FU concentrations, consistent with reduced sensitivity to 5-FU under these conditions. GBM10 cells demonstrated a similar increase in relative BrdU incorporation under physioxia at the lowest concentration tested, whereas GBM001 cells did not exhibit a significant oxygen-dependent difference in response to 5-FU across the concentrations examined. These findings demonstrate that the effects of physioxia on 5-FU response are cell-line dependent. Physiological oxygen tension appears to be associated with reduced 5-FU sensitivity in select GB models. Data are presented as mean ± SEM from three independent biological experiments (n = 3), with technical replicate wells averaged within each independent experiment prior to statistical analysis. Statistical significance was determined by two-way ANOVA with oxygen condition and 5-FU concentration as factors, with * *p* < 0.05 and **** *p* < 0.0001.

To assess the impact of oxygen tension on chemotherapeutic response, GBM001, GBM10, and GBM43 cells were treated with increasing concentrations of 5-fluorouracil (5-FU), a widely used chemotherapy, under normoxic or physioxic conditions. Cell survival was quantified using a BrdU incorporation assay and expressed as percent survival relative to vehicle-treated controls. All three cell lines exhibited a dose-dependent reduction in cell survival in response to 5-FU under both oxygen conditions (Figure 5A–C).

Distinct differences in chemotherapeutic sensitivity emerged under physioxia in a cell line-dependent manner. GBM001 and GBM10 cells did not demonstrate increased survival under physioxia at any concentration of 5-FU tested (Figure 5A,B). This result suggests that physioxia does not universally confer chemoresistance across GB models (Figure 5A). GBM43 cells cultured under physioxia displayed a significant increase in percent cell survival compared to cells grown in normoxia at 10 µM and 50 µM 5-FU, indicating reduced sensitivity to higher drug concentrations (Figure 5C).

These data demonstrate that physioxia can alter GB sensitivity to 5-FU, with the magnitude and direction of this response varying substantially among cell lines. In particular, the increased resistance observed in select GB models suggests that physiological oxygen tension may contribute to a more therapy-tolerant cellular phenotype, whereas the absence of a similar response in other models underscores the context-dependent nature of this effect. Such differential responses to chemotherapy parallel the oxygen-dependent changes in cell-cycle distribution (Figure 3) and signaling pathways (Figure 4) observed earlier in this study. These observations suggest that reduced cell-cycle progression and altered survival-associated signaling may contribute to differences in chemotherapy sensitivity under physioxia. Although our findings do not establish a direct causal relationship between these mechanisms and 5-FU resistance, they identify potential biological processes that warrant further investigation.

When considered with the enhanced migratory capacity and altered cell-cycle dynamics observed under physioxia, these findings support a model in which physiological oxygen tension can shift GB cells toward a phenotype characterized by increased motility and, in select cellular contexts, reduced sensitivity to chemotherapy. The heterogeneous responses observed across these three GB cell lines emphasize that the effects of physioxia are not uniform and may depend on tumor-specific genetic, molecular, and signaling contexts. These findings highlight the importance of incorporating physiologically relevant oxygen conditions into preclinical studies of GB therapeutic response, as conventional atmospheric oxygen culture may fail to capture clinically observed differences in tumor cell behavior and therapeutic vulnerability.

### 3.6. Glioblastoma Cells Maintained Under Continuous Physioxia Exhibit Enhanced Proliferation and Migration

Exposure of primary tumor cells to ambient air can induce rapid and potentially irreversible biological changes [10,11,34]. Notably, work by Broxmeyer and colleagues has shown that brief exposure (minutes) to normoxic conditions can alter transcriptional programs, signaling pathways, and functional behavior of cells that normally reside in low-oxygen niches [11,34]. These findings underscore the importance of maintaining physioxia during tumor cell isolation and culture to preserve native cellular phenotypes.

In the present study, we compared two distinct culture paradigms to assess the impact of continuous physioxia on GB cell behavior. Patient-derived tumor samples AR022 and AR023 were collected, processed, and maintained entirely under physioxic conditions and compared to parallel cultures acclimated to normoxia. In contrast, the GBM001, GBM10, and GBM43 cells used in Figure 1, Figure 2, Figure 3, Figure 4 and Figure 5 were originally derived from the physiological oxygen environment of the brain but were subsequently cultured long-term under ambient air and later re-exposed to conditions of physioxia. This distinction is critical given prior reports that even transient normoxic exposure may permanently reprogram tumor cell behavior.

**Figure 6:**
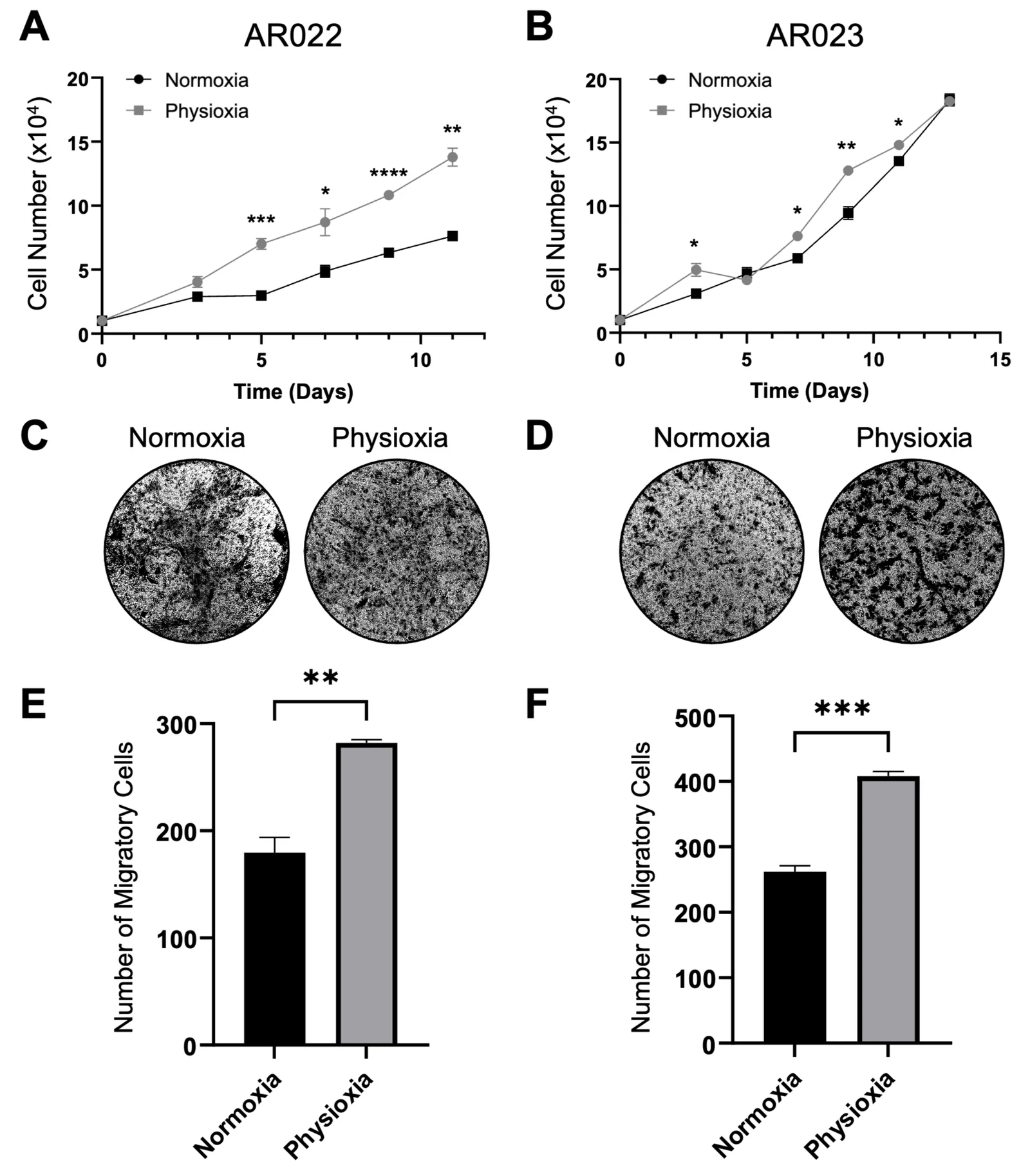
Patient-derived glioblastoma cultures exhibit oxygen-dependent differences in population expansion and migration under physioxia. (**A**,**B**) Longitudinal population expansion of patient-derived GB cultures (**A**) AR022 and (**B**) AR023 was assessed over a 14-day period following establishment and maintenance under physioxic conditions, with cells subsequently cultured under either normoxic (21% O_2_) or physioxic (5% O_2_) conditions. Both AR022 and AR023 cultures demonstrated greater population expansion under physioxia compared with normoxia. Separate wells were harvested at the indicated time points, and viable cell numbers were determined by trypan blue exclusion and hemocytometer counting. (**C**,**D**) Representative images of Transwell migration assays performed with (**C**) AR022 and (**D**) AR023 cells cultured under normoxic or physioxic conditions. (**E**,**F**) Quantification of migrated cells demonstrating significantly increased migratory capacity under physioxia in (**E**) AR022 and (**F**) AR023 cultures. These findings suggest that patient-derived GB cultures maintained under physioxia exhibit increased population expansion and migration compared with cells cultured under normoxia. Data are presented as mean ± SEM from three independent biological experiments (n = 3). Statistical significance was determined using unpaired two-tailed Student’s *t*-tests, with * *p* < 0.05, ** *p* < 0.01, *** *p* < 0.001, and **** *p* < 0.0001.

Analysis of patient-derived AR022 and AR023 cells revealed a significant increase in cell proliferation when maintained under physioxia compared with normoxia-acclimated cultures (Figure 6A,B). In addition to enhanced growth, both AR022 and AR023 cells exhibited increased migratory capacity under physioxic conditions, as evaluated by transwell migration (Figure 6C–F). Notably, the physioxia-induced increase in migration observed in these continuously physioxia-maintained patient-derived cells mirrored the enhanced migratory phenotype seen in GBM001, GBM10, and GBM43 cells under physioxia (Figure 1, Figure 2, Figure 3, Figure 4 and Figure 5), despite differences in culture history.

These findings demonstrate that physiological oxygen tension can substantially influence GB cell behavior and such effects can depend on both cellular context and prior culture history. The enhanced migratory phenotype observed under physioxia was maintained across GB models regardless of whether cells were continuously maintained under physioxia or transitioned from prior normoxic culture, a consistent association between physiological oxygen conditions and increased migratory capacity. Interestingly, the proliferative response to physioxia differed markedly between models. AR022 and AR023 cells, which were maintained under physioxia from the time of isolation and throughout culture, exhibited increased population expansion under physiological oxygen conditions, whereas the established GBM001, GBM10, and GBM43 models displayed reduced population expansion when cultured in physioxia. These divergent responses suggest that cellular culture history and prior oxygen exposure may contribute to the way GB cells respond to subsequent changes in oxygen tension. However, since the models differ in patient background, tumor genetics, isolation procedures, passage history, and culture conditions, these findings cannot establish oxygen history as the sole determinant of the observed differences in proliferation.

Our findings underscore the importance of considering physiological oxygen tension throughout the experimental modeling of GB. The enhanced migratory behavior observed across multiple models, combined with the context-dependent effects of physioxia on population expansion, suggests that conventional atmospheric oxygen culture conditions may influence or obscure clinically relevant GB phenotypes. In particular, the increased population expansion and migration observed in AR022 and AR023 cells maintained under continuous physioxia raises a possible concern: prolonged exposure to atmospheric oxygen during biospecimen processing and cell culture may alter phenotypic states that would otherwise be maintained under physiologically relevant conditions. These findings support the incorporation of controlled physioxic conditions during GB biospecimen processing and downstream experimental applications to better preserve and characterize tumor-associated cellular phenotypes. More broadly, our results highlight the importance of accounting for oxygen tension and culture history when interpreting GB tumor biology, cellular plasticity, and therapeutic response in preclinical models.

## 4. Discussion

GB develops within a physiologically low-oxygen microenvironment that is not accurately reproduced by standard culture conditions. In this study, we show that prolonged culture under physioxia (5% O_2_) alters multiple aspects of GB cell behavior, including migration, proliferation, cell cycle progression, signaling pathway activation, EMT-associated transcription, and therapeutic response. Our findings support a model in which physiological oxygen tension promotes a more invasive and adaptive GB phenotype while differentially regulating proliferation and drug sensitivity, depending on cellular context.

A consistent finding across all GB models examined was enhanced migration under physioxia. GBM001, GBM10, and GBM43 cells all exhibited significantly increased transwell migration despite differences in genetic background, signaling responses, and proliferative behavior. These observations are consistent with previous reports from other patient-derived tumor systems, suggesting that increased motility is a conserved response to physiological oxygen tension. Given the highly infiltrative nature of GB in vivo, these findings suggest that physioxia favors phenotypes associated with invasion and tissue adaptation rather than maximal proliferative output.

In contrast to the apparent increases in migration, physioxia reduced long-term proliferation in GBM001, GBM10, and GBM43 cells. Reduced cell accumulation was associated with increased G0/G1 occupancy and reduced S-phase entry, indicating suppression of cell cycle progression under physiological oxygen conditions. GBM43 cells exhibited the strongest response, including reductions in both S-phase and G2/M populations, which is consistent with a more pronounced cell cycle arrest. Together, these data suggest there is a dichotomy between proliferation and migration in GB cells grown in physioxia, in which invasive behavior is enhanced while proliferative activity is restrained [40]. Such a shift may provide a selective advantage within the brain microenvironment, where adaptation to metabolic stress and therapeutic pressure is required for tumor persistence.

At the molecular level, physioxia increased Slug (SNAI2) expression in all GB lines examined, linking physiological oxygen tension to EMT-associated transcriptional programs. Slug has been implicated in mesenchymal transition, cytoskeletal remodeling, and invasion in GB and other cancers [17]. The consistent induction of Slug across all models suggests that activation of mesenchymal transcriptional programs represents a conserved response to physioxia. In addition to Slug, hypoxia-responsive pathways have been shown to promote GB cell migration through upregulation of ODZ1 (TENM1), a transmembrane protein that enhances actin cytoskeletal remodeling and cell migration downstream of hypoxia-inducible signaling [41]. These observations suggest that activation of mesenchymal and pro-migratory transcriptional programs represents a conserved cellular response to reduced oxygen availability.

Analysis of upstream signaling pathways demonstrated substantial cell line-dependent differences. In GBM001 and GBM10 cells, physioxia increased phosphorylation of PDGFRβ, AKT, and ERK, consistent with activation of signaling pathways associated with GB migration and mesenchymal transition [17]. These findings support a mechanistic relationship between physiological oxygen tension and PDGFRβ-dependent signaling in promoting invasive behavior.

Interestingly, GBM43 cells showed increased Slug expression and migration without detectable increases in PDGFRβ, AKT, or ERK phosphorylation. One explanation for this divergence is the presence of mutant p53 in GBM43 cells. Mutant p53 can constitutively activate the PI3K/AKT and MAPK/ERK signaling pathways. In addition, it enables EMT-associated transcriptional programs to occur independent of receptor tyrosine kinase activation [30,38]. Baseline pathway activation may therefore limit further inducibility under physioxia. Consistent with this interpretation, physioxia increased phospho-p53 (S15) and phospho-β-catenin (S675) levels in GBM43 cells, suggesting engagement of alternative signaling mechanisms linked to invasion and cellular adaptation. Increased β-catenin signaling has previously been associated with EMT-related transcription and invasive behavior in GB [38].

The pronounced cell cycle arrest observed in GBM43 cells may also contribute to the distinct signaling phenotype observed under physioxia. Quiescent GB cells can retain invasive capacity while displaying reduced dependence on growth factor-mediated proliferative signaling [42]. This may explain why GBM43 cells maintained enhanced migration without additional PDGFRβ-associated pathways. Taken together, these findings suggest that although physioxia consistently promotes migration, the signaling pathways supporting this phenotype are strongly influenced by underlying genetic context, including p53 status.

Physioxia also altered therapeutic response in a cell line-dependent manner. GBM43 cells exhibited increased resistance to 5-fluorouracil (5-FU) at higher drug concentrations, while GBM10 cells showed limited resistance only at low-dose treatment. Physioxia did not increase GBM001 cell survival. The observed resistance patterns correlated with reduced proliferation and altered cell cycle progression, consistent with studies linking quiescence and reduced S-phase entry to decreased sensitivity to antimetabolite therapies [42,43,44]. These findings suggest that physiological oxygen tension can promote a therapy-tolerant state in specific GB contexts but does not uniformly induce chemoresistance.

An important observation from this study is the influence of culture history on GB behavior under physioxia. GBM001, GBM10, and GBM43 cells were initially derived from the low-oxygen brain environment but subsequently maintained long-term under ambient air before re-exposure to physioxia. Previous studies by Broxmeyer and colleagues demonstrated that even brief exposure to atmospheric oxygen can induce rapid and potentially irreversible cellular alterations [8,25]. To address this issue, we examined patient-derived AR022 and AR023 cells that were isolated and continuously maintained under physioxia. Compared to normoxia-adapted GB lines, these cells displayed increased proliferation and migration when cultured in physioxic conditions. While enhanced migration was consistent across all models, the increased proliferation observed in continuously physioxic cultures contrasted with the growth suppression seen in normoxia-adapted lines. These findings suggest that prolonged exposure to ambient air may alter GB responses to physiological oxygen tension, particularly with respect to proliferative behavior.

The differential proliferative responses observed between the established GBM001, GBM10, and GBM43 models and the newly derived AR022 and AR023 cultures may reflect differences in prior oxygen exposure. These cell models, however, differ in patient-specific genetic backgrounds, passage history, tumor heterogeneity, isolation procedures, and culture conditions, including serum concentration and growth factor supplementation. Consequently, while these findings are consistent with our hypothesis that prolonged culture under atmospheric oxygen induces persistent phenotypic adaptations, the present study cannot definitively distinguish the contribution of oxygen history from these additional biological and technical variables.

Nevertheless, previous studies have demonstrated that prolonged exposure to atmospheric oxygen induces stable transcriptional, metabolic, and epigenetic alterations that persist after cells are returned to physiological oxygen conditions [10,13,17,20,39]. These data support the possibility that culture history contributes substantially to the observed phenotypes. Future studies using paired patient-derived models maintained continuously under defined oxygen and culture conditions will be necessary to directly isolate the effects of oxygen history.

Our findings demonstrate that physiological oxygen tension promotes an invasive and adaptive GB phenotype characterized by enhanced migration, EMT-associated transcription, altered cell cycle progression, and context-dependent therapeutic resistance. These results suggest that oxygen tension is a major determinant of GB cell state and that conventional normoxic culture conditions may obscure biologically relevant phenotypes. The molecular signature identified in this study differs from that reported in previous investigations of hypoxia-induced GB invasion, which have implicated additional mediators such as ODZ1 (TENM1), a HIF-responsive regulator of actin cytoskeletal remodeling and cell migration, and other canonical hypoxia-induced targets associated with severe oxygen deprivation [13,15,41]. These differences may reflect the distinct oxygen paradigm employed in our study. Whereas many previous investigations have examined acute, severe hypoxia (typically ≤1–2% O_2_ for 24–72 h), we modeled chronic physiological oxygen tension (5% O_2_), which more closely approximates the oxygen environment experienced by normal brain tissue. Short-term exposure to reduced oxygen may not allow tumor cells to fully establish long-term adaptive programs, while prolonged culture under atmospheric oxygen before hypoxic exposure may induce persistent molecular alterations that influence subsequent responses to reduced oxygen tension.

This study highlights the importance of oxygen history as an often-overlooked experimental variable and suggests that oxygen tension during tissue acquisition, cell isolation, expansion, and experimentation can substantially influence the molecular and phenotypic readouts commonly used in cancer biology. Further studies are therefore needed to define how both oxygen concentration and duration of exposure shape GB biology and to establish culture conditions that more accurately model the native tumor microenvironment. By modeling GB under sustained physiological oxygen tension, experimental systems may better recapitulate tumor biology, improve the reproducibility and translational relevance of preclinical studies, and ultimately provide a more accurate platform for evaluating therapeutic responses.

Several important questions emerge from this work. First, future studies should define the precise molecular mechanisms linking physioxia to Slug induction, including the relative contributions of hypoxia-inducible factors, chromatin remodeling, and non-canonical signaling pathways. Second, systematic comparisons of continuously physioxia-maintained versus normoxia-adapted GB cultures will be essential to determine which phenotypes represent true in vivo behavior. Third, given the cell line-specific signaling responses observed here, integrating physioxia modeling with genomic and epigenomic profiling may reveal biomarkers predictive of invasion or therapy resistance. Finally, evaluating standard-of-care therapies, including temozolomide and radiation, under physiological oxygen conditions will be critical for improving the translational relevance of preclinical GB studies.

A limitation of this study is the absence of a healthy, non-tumorigenic astrocyte model as a control for comparison with the GB cell lines. Inclusion of normal astrocytes would have provided an important reference for determining whether the oxygen-dependent changes in proliferation, cell-cycle progression, migration, signaling, and therapeutic response observed in this study are specific to GB cells or represent broader responses of astrocytic cells to physiological oxygen conditions. Since the primary objective of this study was to characterize oxygen-dependent phenotypic plasticity across multiple patient-derived GB models, normal astrocytes were not included in the current experimental design. Future studies incorporating healthy astrocytes or other non-tumorigenic neural cell models will be important for defining the tumor-specificity of these responses and determining whether physiological oxygen conditions selectively influence malignant versus normal cellular phenotypes.

In summary, our study demonstrates that physioxia is a central regulator of GB cell behavior, shaping migration, proliferation, signaling, and therapeutic response. Incorporating physioxia into experimental design will be essential for accurately modeling GB biology and for developing more effective therapeutic strategies.

## 5. Conclusions

This study demonstrates that sustained physiological oxygen tension is an important regulator of GB cell state and behavior. By modeling chronic physioxia at 5% O_2_, we show that oxygen tension influences multiple interconnected features of GB biology, including migration, population expansion, cell-cycle progression, mesenchymal-associated transcription, intracellular signaling, and response to chemotherapy. Importantly, the effects of physioxia were not uniformly growth-promoting or growth-suppressive. Rather, physiological oxygen tension consistently enhanced migratory capacity while producing context-dependent effects on proliferation and therapeutic response. These findings support a model in which GB cells can adopt a more motile and adaptive phenotype under physiological oxygen conditions, potentially allowing tumor cells to balance invasive capacity with reduced proliferative activity within the brain microenvironment.

A major finding of this study is the consistent enhancement of GB cell migration under physioxia across genetically distinct established GB models and patient-derived cultures. This pro-migratory phenotype was accompanied by increased SNAI2 (Slug) expression across all models, supporting activation of transcriptional programs associated with cellular plasticity and invasion. However, the signaling mechanisms associated with this phenotype were not uniform. GBM001 and GBM10 demonstrated increased PDGFRβ, AKT, and ERK phosphorylation under physioxia, whereas GBM43 maintained enhanced migration without corresponding increases in these pathways and instead demonstrated changes in mutant p53- and β-catenin-associated signaling. These findings emphasize that physiological oxygen tension can produce a shared functional phenotype through distinct molecular mechanisms, highlighting the substantial biological heterogeneity of GB.

Our findings also reveal an important relationship between oxygen tension, proliferation, and therapeutic response. In established GBM001, GBM10, and GBM43 cultures, physioxia reduced population expansion and increased G0/G1 accumulation with reduced S-phase entry, consistent with a shift away from active proliferation. In parallel, physioxia increased resistance to 5-FU in select cell models, although this effect was not universal. These observations suggest that reduced proliferative activity and altered survival-associated signaling under physiological oxygen tension may contribute to a therapy-tolerant cellular state in specific GB contexts. Importantly, the absence of increased 5-FU resistance in GBM001 demonstrates that physioxia should not be viewed as a universal driver of chemoresistance, but rather as an environmental factor whose effects depend on tumor-specific molecular context.

An important and innovative aspect of this study is the consideration of oxygen history as an experimental variable in GB research. Established GB models that had experienced prolonged culture under atmospheric oxygen before re-exposure to physioxia exhibited reduced population expansion under 5% O_2_, whereas patient-derived AR022 and AR023 cultures that were isolated and maintained under continuous physioxia demonstrated increased population expansion and migration under physiological oxygen conditions. Although differences in genetic background, passage history, isolation procedures, and culture conditions prevent oxygen history from being established as the sole explanation for these divergent proliferative responses, the findings raise the important possibility that prolonged exposure to atmospheric oxygen can influence subsequent tumor cell behavior. In contrast, enhanced migration under physioxia was observed across both culture paradigms, supporting a more consistent association between physiological oxygen tension and the invasive phenotype.

The novelty of this work therefore extends beyond demonstrating that oxygen affects GB cell behavior. Rather, this study emphasizes how the duration and history of oxygen exposure influence the phenotypic state being measured. Much of the existing literature examining oxygen-dependent GB biology has focused on acute or severe hypoxia, whereas this study specifically models sustained physiological oxygen tension at 5% O_2_. This approach provides a complementary perspective on oxygen-dependent GB plasticity by examining conditions that more closely approximate the oxygen environment of normal brain tissue. Our findings suggest that chronic physioxia may reveal phenotypes that are not apparent under conventional atmospheric oxygen culture and that oxygen exposure during tumor isolation, cell expansion, and experimental manipulation may represent an important source of biological variability in preclinical GB studies.

Collectively, these findings establish oxygen tension as an active component of the GB microenvironment rather than simply a technical parameter of cell culture. The consistent enhancement of migration, combined with context-dependent effects on proliferation, signaling, and chemotherapy response, demonstrates that physiological oxygen conditions can substantially alter the cellular state used to model GB. These observations have important implications for the interpretation and reproducibility of preclinical studies, particularly those investigating tumor invasion, cellular plasticity, and therapeutic resistance. Incorporating controlled physioxic conditions throughout GB biospecimen processing, culture, and experimentation may therefore improve the physiological relevance of experimental models and help preserve tumor-associated phenotypes that may be altered by prolonged exposure to atmospheric oxygen.

Future studies should determine the molecular mechanisms responsible for physioxia-induced Slug expression and define how oxygen history interacts with tumor genotype, including p53 status, to determine GB cell state. Systematic paired studies using patient-derived cultures maintained continuously under defined oxygen conditions will be particularly important for distinguishing effects caused by oxygen history from those arising from intrinsic tumor heterogeneity. In addition, evaluating standard-of-care treatments such as temozolomide and radiation under physiological oxygen conditions will be important for determining whether the oxygen-dependent phenotypes identified here influence clinically relevant therapeutic responses.

In conclusion, our findings demonstrate that chronic physiological oxygen tension regulates GB plasticity across multiple dimensions of tumor biology, with a particularly consistent effect on migratory behavior and more context-dependent effects on proliferation and therapeutic sensitivity. By incorporating both physiological oxygen tension and oxygen history into experimental design, this study provides a framework for more accurately modeling the GB microenvironment and highlights an underappreciated source of variation in preclinical cancer research. Ultimately, physiologically relevant oxygen modeling may improve the biological fidelity, reproducibility, and translational value of GB experimental systems and provide a more appropriate platform for identifying vulnerabilities that can be therapeutically exploited.

## Data Availability

The data presented in this study are available from the corresponding author upon reasonable request.

## Supplementary Materials

The following supporting information can be downloaded at: https://www.mdpi.com/article/10.3390/cancers18162620/s1.
